# Histopathological stratification of primary aldosteronism using the CYP11B2 size ratio: first evaluation in clinical routine

**DOI:** 10.1111/his.70254

**Published:** 2026-08-04

**Authors:** Adam Stenman, Tim Nilsson, Henrik Falhammar, C. Christofer Juhlin

**Affiliations:** ^1^ Department of Breast, Endocrine Tumors and Sarcoma Karolinska University Hospital Stockholm Sweden; ^2^ Department of Molecular Medicine and Surgery Karolinska Institutet Stockholm Sweden; ^3^ Department of Endocrinology Karolinska University Hospital Stockholm Sweden; ^4^ Department of Oncology‐Pathology Karolinska Institutet Stockholm Sweden; ^5^ Department of Pathology and Cancer Diagnostics Karolinska University Hospital Stockholm Sweden

## Abstract

**Background:**

In primary aldosteronism (PA), the HISTALDO framework classifies aldosterone‐producing lesions as ‘classical’ (solitary nodule) or ‘non‐classical’ (multinodular or diffuse) based on CYP11B2 immunohistochemistry, but interpretation is complicated by frequent micronodules, even in patients without PA. The CYP11B2 size ratio (B2R), a quantitative measure of size disproportion between CYP11B2‐positive nodules, offers an objective way to assess lesion architecture and has shown value in predicting cure versus relapse. In this study, diagnostic classification was intentionally B2R‐guided, generating a modified HISTALDO B2R system in clinical routine.

**Objective:**

Prospective evaluation of the prognostic value of B2R‐guided classification in routine pathology and assessment of postoperative outcomes across B2R‐defined subgroups.

**Methods:**

Specimens of 70 adrenalectomies underwent multisection CYP11B2 staining; 61 patients had at least 6 months of follow‐up (mean age 53 years, 54% were females). All had adrenal vein sampling and confirmatory testing before unilateral laparoscopic adrenalectomy. Imaging showed unilateral findings in 51%, bilateral in 8% and no detectable nodules in 41%. A B2R ≥8.1 defined classical lesions; B2R < 8.1 non‐classical. Outcomes at 6–12 months were assessed using PASO criteria, which classify biochemical and clinical success as absent, partial or complete.

**Results:**

Biochemical cure was achieved in 95% of patients with classical B2R morphology, compared with 40% of those with non‐classical B2R morphology (*p* < 0.001). Complete clinical success was also significantly more frequent in the classical B2R group than in the non‐classical group (57% versus 25%, *p* = 0.0235). Medication‐based measures supported these findings: patients with classic histology showed a trend towards reductions in antihypertensive medication expressed as defined daily doses (DDD) (median ΔDDD 2.83 versus 2.00, *p* = 0.1254), and a significantly greater reduction in potassium supplementation (median ΔDDD 1.25 versus 0.00, *p* < 0.001) compared to those with non‐classical histology.

**Conclusions:**

B2R‐guided HISTALDO yields biologically distinct subgroups with clear prognostic separation. Earlier work using the original HISTALDO definition reported ~40%–45% persistent biochemical aldosteronism in non‐classical cases. Our findings reproduce this poor non‐classical outcome profile in a larger and more consistently defined B2R‐classified cohort. Importantly, incorporating B2R reduces misclassification of tumours as non‐classical when background nodularity is present despite a dominant CYP11B2‐positive lesion.

## Graphical abstract

CYP11B2 immunohistochemistry identifies aldosterone‐producing nodules and enables objective subtyping using the B2 ratio (B2R), defined as the size ratio between the largest and second‐largest CYP11B2‐positive nodules. A B2R ≥8.1 indicates classical histology with a dominant lesion and high likelihood of cure, whereas B2R <8.1 reflects non‐classical multinodular disease associated with lower cure rates and increased risk of recurrence.

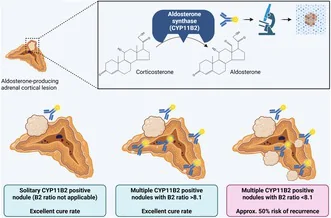

**Abbreviations**
ARR: Aldosterone–renin ratio
AVS: adrenal venous sampling
CT: computed tomography
IQR: interquartile range
MAPN/MAPMN: multiple aldosterone‐producing nodules or micronodules
PA: primary aldosteronism
PASO: Primary Aldosteronism Surgery Outcome
SD: standard deviation

## Introduction

Hypertension affects 32% of women and 34% of men aged 30–79 worldwide.^1^ The prevalence of primary aldosteronism (PA) has been estimated at 5.9% of all patients with hypertension in primary care and 11.8% of patients with stage 3 hypertension (defined as a blood pressure of 180/110 mmHg or higher).^2^ Hypertension caused by PA is associated with an increased risk of stroke, coronary artery disease and heart failure compared to essential hypertension.^3^

The risk of complications that follow hypertension in general and PA in particular warrants evidence‐based treatment and follow‐up regimes. Aldosterone–renin ratio (ARR) is used to screen for PA, and the salt loading test or fludrocortisone suppression test can be used to confirm the diagnosis if necessary.^4^, ^5^, ^6^ To decide if PA is unilateral or bilateral, adrenal venous sampling (AVS) has traditionally been performed if adrenalectomy is a treatment option.^6^ The more severe PA, the higher the chance of unilateral disease.^7^ Laparoscopic unilateral adrenalectomy is the gold‐standard treatment for unilateral disease with a possibility of cure.^8^ Bilateral disease, or unilateral cases not suited for surgery, is treated with mineralocorticoid receptor antagonists (MRA); the goal is not to cure, but to reduce symptoms and prevent complications.^8^, ^9^ MRA treatment can also be used while patients are awaiting surgery to control blood pressure and reduce hypokalaemia, and preoperative use of MRA is associated with better biochemical outcomes compared to surgery without preoperative MRA.^10^ Surgical outcomes are evaluated using the Primary Aldosteronism Surgery Outcome (PASO) assessment, which classifies biochemical and clinical success as absent, partial or complete.^11^

HISTALDO (histopathology of primary aldosteronism) is a World Health Organization–endorsed system for classifying adrenal lesions in patients with PA.^12^ This algorithm relies on CYP11B2 (aldosterone synthase) immunohistochemistry to identify aldosterone‐producing nodules. Within HISTALDO, aldosterone‐producing adenomas (solitary lesions ≥1 cm) and aldosterone‐producing nodules (solitary lesions <1 cm) are classified as ‘classical histology’, a category associated with excellent cure rates following unilateral adrenalectomy. In contrast, multiple aldosterone‐producing nodules or micronodules (MAPN/MAPMN) and diffuse hyperplasia of the zona glomerulosa are classified as non‐classical histology and are associated with an almost 50% risk of relapse in the contralateral adrenal gland.

However, many normal adrenal glands also contain aldosterone‐producing micronodules, and the number of these lesions has been shown to increase with age.^13^, ^14^, ^15^, ^16^, ^17^, ^18^ Because these individuals do not have PA, the presence of CYP11B2‐positive nodules does not appear to correlate strongly with disease. This suggests that small CYP11B2‐positive nodules may be unrelated to PA itself and may therefore complicate pathological interpretation.

To address this issue, a CYP11B2 size ratio (B2R), defined as the ratio between the diameter of the largest and second‐largest nodules in a CYP11B2‐stained specimen, has been introduced.^13^, ^19^ A B2R of ≥8.1 is used to classify a lesion as classical HISTALDO despite the presence of small background lesions, whereas a B2R of <8.1 supports classification as non‐classical histology^19^ (Figure 1). This modification was shown to alter the original HISTALDO classification by reclassifying some specimens initially categorized as non‐classical into the classical histology group, specifically aldosterone‐producing adenomas, and this revised subtyping also improved correlations to patient outcome.^19^ However, the proposed B2R modification of HISTALDO has only been applied retrospectively and has not yet been evaluated in routine clinical practice.^13^, ^19^, ^20^ This study aimed to evaluate whether a B2R‐modified HISTALDO classification can serve as a practical and prognostically meaningful method to separate classical and non‐classical lesions from patients with PA in routine clinical practice.

**Figure 1:**
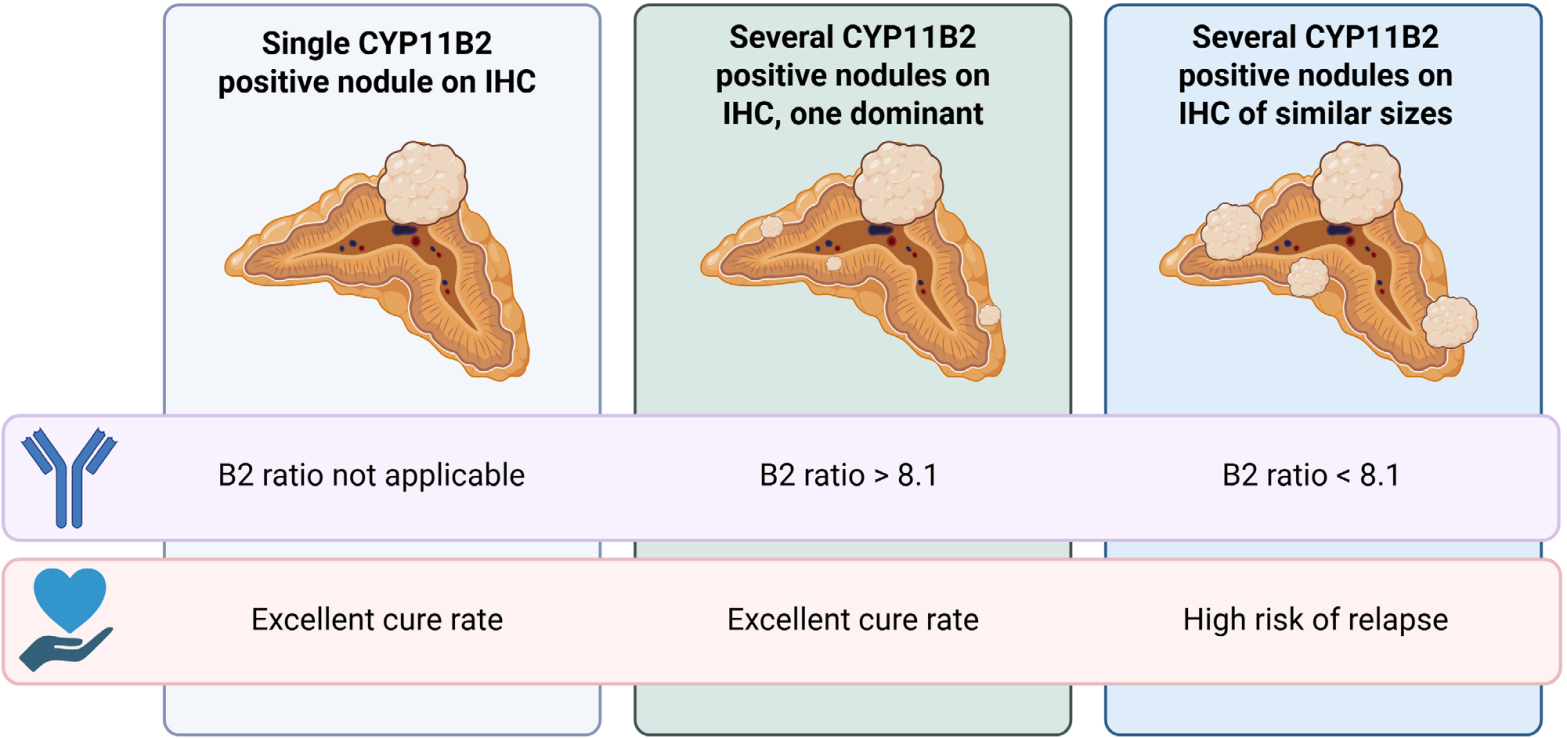
The B2 ratio as an adjunct tool to subtype primary hyperaldosteronism. Three prototypical examples are shown. Left: a single CYP11B2‐positive nodule identified on immunohistochemistry, for which the B2 ratio is not applicable, is associated with an excellent cure rate after adrenalectomy. Middle: several CYP11B2‐positive nodules are present, but one dominant nodule is substantially larger than the others, resulting in a B2 ratio >8.1 and indicating a classical histological pattern with an excellent cure rate. Right: several CYP11B2‐positive nodules of similar size are identified, resulting in a B2 ratio <8.1 and indicating a non‐classical histological pattern associated with a high risk of relapse. Created using BioRender.com.

## Methods

### Patients

This prospective study investigated the use of a B2R‐modified HISTALDO system in clinical routine practice in patients with unilateral PA. Patients who underwent a laparoscopic unilateral adrenalectomy at Karolinska University Hospital in Stockholm, Sweden between January 2023 and August 2025 were included (*n* = 70). Inclusion criteria were biochemically confirmed PA with an unstimulated AVS lateralization index >4, informed consent before surgery, laparoscopic unilateral adrenalectomy, and a minimum of 6 months follow‐up. Patients with PA were diagnosed according to current Swedish national guidelines for adrenal tumours. After presenting a pathologically high ARR, patients were subjected to confirmatory testing. All patients underwent a computed tomography (CT) of the adrenal glands and AVS.

Pseudonymized clinical data were collected from the patients' electronic medical records; including age, sex, height, weight, body mass index (BMI), surgery date, histopathological diagnosis, CT findings, confirmatory test, B2R and AVS index. Preoperative and postoperative blood pressure and laboratory values for potassium, renin and aldosterone concentrations were recorded. Defined daily dose (DDD) of potassium supplementation and antihypertensive medication was calculated according to the World Health Organization (WHO) Collaborating Centre (atcddd.fhi.no).

Following adrenalectomy, patient outcomes were assessed by a multidisciplinary team including two study authors (AS, HF), according to the PASO criteria, evaluating both clinical and biochemical parameters as proposed. Patients were required to have at least 6–12 months documented follow‐up. Clinical success was classified as complete (normotension without antihypertensive medication), partial (improvement in blood pressure and/or reduction in antihypertensive medication) or absent (no improvement in blood pressure and/or no reduction in antihypertensive medication). Biochemical success was classified as complete (normal serum potassium without supplementation and normal ARR), partial (normal serum potassium without supplementation and improved but abnormal ARR) or absent (persistent hypokalaemia and/or persistently elevated ARR).

The study has been approved by the Swedish National Ethical Authority and informed consent had been obtained.

### Histopathology and Immunohistochemistry

Adrenal tissue specimens were processed according to a standardized histopathological workflow at the Department of Pathology and Cancer Diagnostics, Karolinska University Hospital, referred to as the ‘Karolinska protocol’,^13^ summarized in Table 1. For glands containing a single macroscopically visible adrenocortical nodule, representative sections of the lesion were sampled together with at least five additional cassettes of macroscopically normal adrenal tissue. The visible lesion and any additional nodules identified on subsequent microscopic examination were subjected to CYP11B2 immunohistochemistry. If no additional microscopic nodules were identified, three randomly selected slides from histologically normal adrenal tissue were also stained. In glands with multiple macroscopically visible nodules, representative sections from each nodule were obtained and stained individually. In the absence of macroscopic nodules, the adrenal gland was entirely embedded and submitted for histopathological examination. Any nodules identified microscopically were stained for CYP11B2, together with four additional slides from morphologically unremarkable areas. Importantly, the protocol required CYP11B2 analysis of all nodules identified either macroscopically or microscopically.

**Table 1:** The ‘Karolinska protocol’ for detection of clinically relevant CYP11B2‐positive nodules and micronodules

Macroscopic finding	Histology sampling	CYP11B2 immunohistochemical staining
One macroscopic nodule	Sample the visible nodule and ≥5 blocks of grossly normal adrenal tissue	Stain the dominant nodule. If no additional microscopic nodules are found, stain 3 random normal slides. If microscopic nodules are identified, stain each one
Several macroscopic nodules	Sample every macroscopic nodule	Perform CYP11B2 staining on each macroscopic nodule
No macroscopic nodules	Embed the entire adrenal gland	Stain every microscopic nodule plus 4 additional slides from morphologically normal adrenal tissue

To ensure consistency in macroscopic handling, all resected adrenal glands from patients with PA were grossed by an endocrine pathologist. Immunohistochemical analyses were performed as previously reported using a standardized protocol on the automated Ventana BenchMark ULTRA platform.^13^ Formalin‐fixed, paraffin‐embedded tissue blocks were sectioned at 4 μm, deparaffinized and subjected to heat‐induced antigen retrieval using Ultra CC1 buffer at 100°C. Endogenous peroxidase activity was inhibited before incubation with the primary anti‐CYP11B2 antibody (mouse monoclonal, clone 41‐17B) for 32 min at room temperature. Detection was performed using HRP Multimer and DAB, followed by haematoxylin counterstaining and blueing. All stained slides were interpreted by a single endocrine pathologist (CCJ) to reduce interobserver variability.

The CYP11B2 size ratio (B2R) was calculated as the ratio between the maximum diameters of the largest and second‐largest CYP11B2‐positive nodules found. Measurements were obtained from digitally scanned slides using calibrated software. Based on the previously proposed cut‐off of 8.1, cases with a B2R of ≥8.1 were categorized as B2R classical histology, whereas cases with a B2R of <8.1 were categorized as B2R non‐classical histology.^13^, ^19^

Following the primary analyses, an exploratory pilot comparison was performed in a randomly selected subset of 15 adrenal glands to evaluate whether a simplified CYP11B2 sampling strategy would yield similar B2R classifications. The simplified approach consisted of a single CYP11B2 stain of the dominant lesion (or one representative section in glands without a macroscopically visible nodule). The resulting classifications were compared with those obtained using the full Karolinska protocol. As this analysis was performed post hoc, it was considered exploratory and was not a predefined study objective.

### Statistical Analyses

Continuous variables (age, BMI, blood pressure, aldosterone, renin, potassium, DDD, B2 ratio and the AVS lateralization index) were presented as mean with standard deviation (SD) or median and interquartile range (IQR), as appropriate. Categorical variables (sex, hypokalaemia, HISTALDO classification, PASO classifications, imaging findings, and surgical technique) were presented as counts and percentages.

Continuous variables such as doses of antihypertensive medication and potassium supplementation were analysed with the Mann–Whitney *U* test and presented as ΔDDD. The analysis used asymptotic *p*‐values due to ties in the data. Categorical variables such as biochemical and clinical success were analysed using Fisher's exact test. The selection of tests was made with consideration for the small cohort size. Comparisons were performed according to the B2R‐modified HISTALDO classification of classical and non‐classical histology. Statistical analyses were performed using R version 4.5.2. Statistical significance was defined as *p* < 0.05.

## Results

### Baseline Characteristics

Baseline characteristics of the cohort are summarized in Table 2. A total of 70 patients underwent laparoscopic unilateral adrenalectomy and were assessed for B2R‐guided HISTALDO, and 61 with sufficient postoperative follow‐up were included. The mean age was 52.8 ± 9.9 years, and 54% were female. Median BMI was 27.3 kg/m^2^ (IQR 6.8). Mean preoperative blood pressure was 142.4 ± 19.9 mm Hg systolic and 90.7 ± 13.5 mm Hg diastolic.

**Table 2:** Baseline characteristics of patients with primary aldosteronism having unilateral adrenalectomy

Variables	Value
Age	
Mean (SD)	52.8 (9.9)
Sex	
Female	33 (54%)
Male	28 (46%)
BMI	
Median (IQR) (kg/m^2^)	27.3 (6.8)
Systolic blood pressure	
Mean (SD) (mmHg)	142.4 (19.9)
Diastolic blood pressure	
Mean (SD) (mmHg)	90.7 (13.5)
Antihypertensive medication (DDD)	
Median (IQR)	3 (3)
Potassium supplementation (DDD)	
Median (IQR)	0.25 (1)
Serum potassium (K+)	
Mean (SD) (mmol/l)	3.5 (0.4)
Hypokalaemia S‐Potassium <3.5 mmol/l (yes/no)	
Yes	28 (46%)
No	33 (54%)
Imaging	
Unilateral nodules	31 (51%)
Bilateral nodules	5 (8%)
No nodules	25 (41%)
AVS LI	
Median (IQR)	14 (14.7)
Surgical technique	
Laparoscopic unilateral adrenalectomy	61 (100%)

Clinical parameters are shown as mean (SD), *n* (%) and median (IQR), as appropriate.

AVS LI, adrenal venous sampling lateralization index; BMI, body mass index; DDD, defined daily dose.

Patients required substantial medical treatment preoperatively, with a median antihypertensive drug burden of 3.0 DDD (IQR 3.0) and potassium supplementation of 0.25 DDD (IQR 1.0). Mean serum potassium was 3.5 ± 0.4 mmol/l, and 46% of patients had hypokalaemia (serum potassium <3.5 mmol/l).

On imaging, 51% of patients had unilateral adrenal nodules, 8% had bilateral nodules and 41% had no visible nodules. Median lateralization index at AVS was 14.0 (IQR 14.7). Median preoperative serum aldosterone concentration was 729 pmol/l (IQR 528). All patients underwent laparoscopic unilateral adrenalectomy.

### Postoperative Biochemical and Clinical Outcomes

Postoperative outcomes stratified by histological subtype are presented in Table 3 and detailed according to PASO criteria in Table 4.

**Table 3:** Postoperative outcomes in patients with primary aldosteronism according to PASO criteria and changes in DDD for antihypertensive medication and potassium supplementation

Outcomes	B2R classical (*n* = 21)	B2R non‐classical (*n* = 40)	*P*‐value
Biochemical cure, *n* (%)	20 (95%)	16 (40%)	**<0.001**
Clinical cure, *n* (%)	12 (57%)	10 (25%)	**0.0235**
ΔDDD BT, median (IQR)	2.83 (2.00)	2.00 (2.83)	0.1254
ΔDDD K, median (IQR)	1.25 (1.25)	0.00 (0.56)	**<0.001**

Parameters shown as n (%) or median (IQR), as appropriate. *p* ≤0.05 were considered statistically significant.

BT, antihypertensive medication; DDD, defined daily dose; K, potassium supplementation.

**Table 4:** Postoperative outcomes in patients with primary aldosteronism according to PASO specified with the level of success

Outcomes	B2R classical (*n* = 21)	B2R non‐classical (*n* = 40)	*P*‐value
Biochemical outcomes			**<0.001**
Complete	20 (95%)	16 (40%)	
Partial	1 (5%)	12 (30%)	
Absent	0 (0%)	12 (30%)	
Clinical outcomes			**0.0159**
Complete	12 (57%)	10 (25%)	
Partial	9 (43%)	23 (58%)	
Absent	0 (0%)	7 (17%)	

Parameters are shown as *n* (%).

Patients with B2R classical histology (Figure 2) (*n* = 21) demonstrated significantly superior biochemical outcomes compared with those with B2R non‐classical histology (Figure 3) (*n* = 40). Biochemical cure was achieved in 95% of B2R classical cases compared with 40% in the B2R non‐classical group (*p* < 0.001).

**Figure 2:**
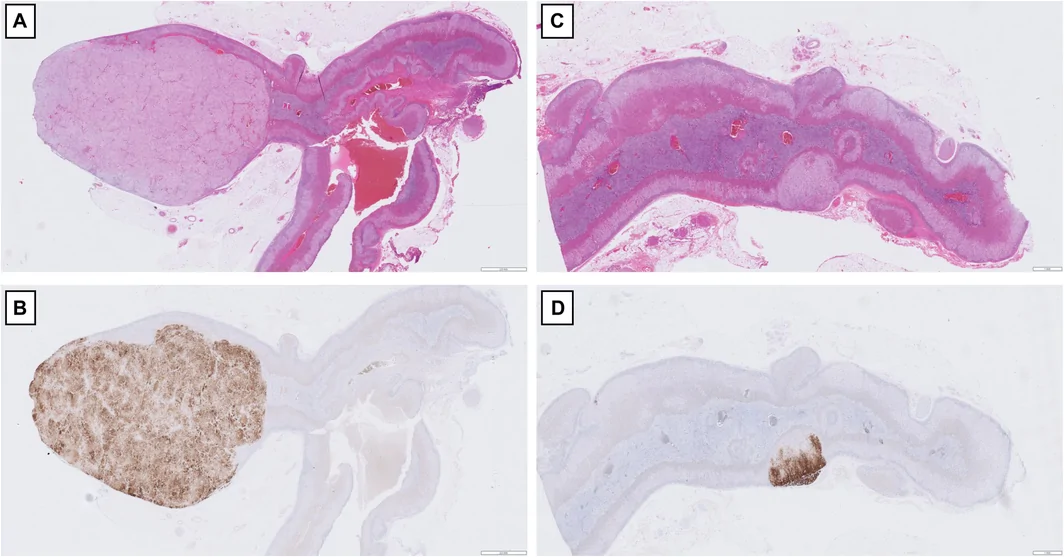
Representative resected adrenal specimen with classical histology and a B2 ratio of 9. The patient achieved biochemical and clinical cure according to PASO criteria. (**A**) Haematoxylin and eosin (H&E) stain demonstrating a macroscopically visible nodule, measuring 18 mm. (**B**) CYP11B2 immunohistochemistry confirmed aldosterone production in this nodule. (**C**) H&E stain of an additional section from the same adrenal gland showing a second 2 mm nodule, also identified at gross examination. (**D**) CYP11B2 immunoreactivity confirmed this second nodule to be aldosterone‐producing.

**Figure 3:**
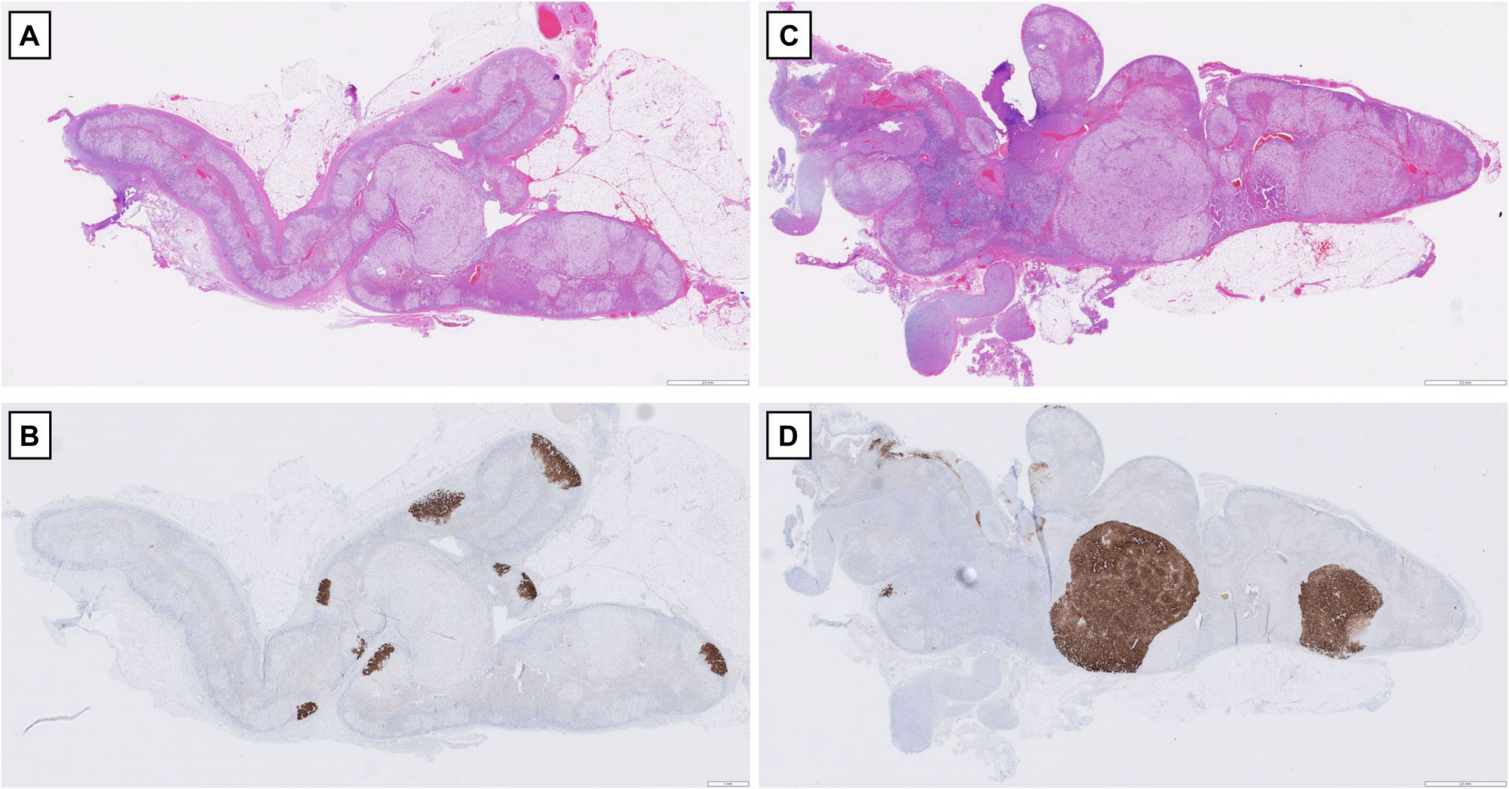
Representative resected adrenal specimen with non‐classical histology and a B2 ratio of 1. The patient achieved only partial biochemical cure according to PASO criteria. (**A**) Haematoxylin and eosin (H&E) stain showing several macroscopically visible nodules of varying sizes. (**B**) CYP11B2 immunohistochemistry demonstrated aldosterone production in several smaller nodules, whereas the two largest nodules in this section were negative, underscoring the importance of immunohistochemistry for functional assessment. (**C**) H&E stain of an additional section from the same adrenal gland revealing additional cortical nodules. (**D**) CYP11B2 immunoreactivity confirmed these larger nodules to be aldosterone‐producing. [Colour figure can be viewed at wileyonlinelibrary.com]

Rates of complete clinical success did differ significantly between groups, occurring in 57% of B2R classical and 25% of B2R non‐classical patients (*p* = 0.0235).

In a randomly selected subset of 15 adrenal glands, the B2R classification obtained using the full Karolinska protocol was compared with that obtained using a simplified strategy based on a single CYP11B2‐stained section. Six cases (40%) would have been reclassified by the simplified approach, all from non‐classical to classical morphology, whereas no cases changed in the opposite direction.

### PASO‐Defined Outcomes

According to PASO criteria (Table 4), complete biochemical success was the predominant outcome among patients with B2R classical histology (95% complete, 5% partial, 0% absent). In contrast, biochemical outcomes in the B2R non‐classical group were more heterogeneous, with 40% complete, 30% partial and 30% absent biochemical success.

For clinical outcomes, patients with B2R classical histology achieved complete clinical success in 57% and partial success in 43%, with no cases of absent clinical success. Among B2R non‐classical patients, 25% achieved complete clinical success, 58% partial success and 17% showed absent clinical success.

### Changes in Antihypertensive Therapy and Potassium Supplementation

Medication‐based outcome measures supported the biochemical findings (Table 3). Patients with B2R classical histology experienced a reduction in antihypertensive treatment burden, with a median decrease of 2.83 DDD (IQR 2.00), compared to 2.00 (IQR 2.83) DDD in the B2R non‐classical group; although this difference did not reach statistical significance (*p* = 0.1254).

In contrast, the reduction in potassium supplementation was significantly greater in the B2R classical group (ΔDDD 1.25, IQR 1.25) compared with the B2R non‐classical group (ΔDDD 0.00, IQR 0.56, *p* < 0.001).

## Discussion

In this prospective study, we analysed data from 61 patients with PA who were classified using the B2R‐modified HISTALDO system. The study aimed to evaluate the prognostic value and clinical and biochemical outcomes in subgroups of the B2R modification in clinical routine practice. Patients with B2R ‘classical’ histology were significantly more likely to achieve complete biochemical success compared to B2R ‘non‐classical’ histology.

The difference found between the B2R classical and B2R non‐classical groups is consistent with the original HISTALDO classification.^21^ This study shows a greater difference between the groups and more homogeneous subgroups with regard to biochemical success. The high rate of biochemical cure in the B2R‐modified classical group suggests that there is a dominant disease‐causing nodule which was removed during surgery.^13^, ^19^ It further supports previous studies indicating that the risk of recurrence is high in the B2R‐modified non‐classical group where the ratio is small, implying multifocal disease with approximately one third of patients not achieving biochemical success following surgery.^13^, ^19^

The difference in clinical success was also statistically significant, but the chance of complete clinical success was almost double in the B2R classical group. The fact that biochemical and clinical success do not necessarily correlate is well known and is possibly associated with the multifactorial nature of hypertension.^22^, ^23^, ^24^ Patients may have both essential hypertension and secondary hypertension due to PA and therefore still have essential hypertension despite being cured of their PA. Moreover, long‐standing primary aldosteronism may lead to partially irreversible vascular and renal changes, thereby increasing the likelihood of persistent hypertension after correction of aldosterone excess.^22^, ^23^, ^24^, ^25^

The microscopic investigation of PA specimens using CYP11B2 immunohistochemistry can be complicated by incidental nodular findings which have been shown to exist in many adrenal specimens.^14^, ^15^, ^16^, ^17^, ^18^ Indeed, the implementation of the B2R‐modified HISTALDO classification was driven mainly by this dilemma and constructed to aid the distinction between classical histology with incidental nodular background activity and true non‐classical histology. The B2R modification yields more homogeneous subgroups with prognostic separation which could be used in clinical practice in order to provide more evidence‐based follow‐up regimes and prognosticates, and the algorithm has been reproduced by others.^13^, ^19^, ^20^

Limitations need to be acknowledged. This was a single‐centre study at a tertiary referral centre with a relatively small cohort size. Only patients who were assessed for surgical treatment were included, and we have no data on how they differ from the patients who were assigned medical treatment due to bilateral disease at diagnosis. Outcomes were assessed using PASO criteria at 6–12 months of follow‐up, and no long‐term follow‐up data were used in the study. Moreover, the discrepancy between the modest rate of unilateral nodules on imaging and the strong AVS lateralization is especially important for this study. This suggests enrichment for cases in which the lateralized aldosterone source was not radiologically obvious, potentially affecting the relative frequency of classical versus non‐classical histopathological patterns and limiting direct comparison with cohorts dominated by radiologically apparent unilateral adenomas. Another limitation is that the Karolinska protocol (Table 1) is relatively resource‐intensive and may not be feasible in all pathology settings. In an exploratory pilot comparison, a simplified CYP11B2 sampling strategy reclassified 40% of cases from non‐classical to classical morphology, suggesting that limited sampling systematically under‐detects additional aldosterone‐producing micronodules. Future studies should therefore investigate whether the protocol can be streamlined without compromising diagnostic accuracy.

In conclusion, we find that using a B2R‐modified HISTALDO system yields valuable prognostic information and distinct subgroups. Incorporating the modification into routine clinical practice might provide a reproducible and simple tool for pathologists to use, as well as a model that aids in postoperative risk stratification.

## Author contributions

CCJ conceived and designed the study. AS and HF analysed and interpreted the patient data according to the PASO criteria. AS and TN performed the statistical analyses. CCJ conducted the histological examinations and prepared the figures. AS, TN and CCJ drafted the manuscript. All authors reviewed, revised and approved the final manuscript.

## Funding information

This work was supported by Stockholm County Council and Karolinska Institutet (HF), and the Cancer Research Foundations of Radiumhemmet (CCJ).

## Conflicts of interest statement

The authors declare no conflicts of interest.

## Data Availability

The data that support the findings of this study are available on request from the corresponding author. The data are not publicly available due to privacy or ethical restrictions.
